# The validity and IRT psychometric analysis of Chinese version of Difficult Doctor-Patient Relationship Questionnaire (DDPRQ-10)

**DOI:** 10.1186/s12888-023-05385-5

**Published:** 2023-12-01

**Authors:** Jianhua Du, Yufei Wang, Aoxue Wu, Yinan Jiang, Yanping Duan, Wenqi Geng, Lin Wan, Jiarui Li, Jiaojiao Hu, Jing Jiang, Lili Shi, Jing Wei

**Affiliations:** 1 Department of Psychological Medicine, Peking Union Medical College Hospital, Chinese Academy of Medical Sciences & Peking Union Medical College, Beijing, China; 2 4+4 Medical Doctor Program, Chinese Academy of Medical Sciences & Peking Union Medical College, Beijing, China; 3 Eight-Year Medical Doctor Program, Chinese Academy of Medical Sciences & Peking Union Medical College, Beijing, China

**Keywords:** Doctor-patient relationship, Patient safety, Item response theory, DDPRQ-10, Validity

## Abstract

**Objective:**

The doctor-patient relationship (DPR) plays a crucial role in the Chinese healthcare system, functioning to improve medical quality and reduce medical costs. This study examined the psychometric properties of the Chinese version of the Difficult Doctor-Patient Relationship Questionnaire (DDPRQ-10) among general hospital inpatients in China.

**Methods:**

The research recruited 38 resident doctors responsible for 120 participants, and factor analyses were used to assess the construct validity of the scale. Convergent validity was evaluated by examining the correlation between DDPRQ-10 and depressive symptoms, burnout, and self-efficacy, using the Patient Health Questionnaire Depression Scale-9 item (PHQ-9), and the Maslach Burnout Inventory (MBI). Both multidimensional item response theory (MIRT) and unidimensional item response theory (IRT) frameworks were used to estimate the parameters of each item.

**Results:**

The Chinese version of DDPRQ-10 showed satisfactory internal consistency (Cronbach's alpha = 0.931), and fitted in a modified two-factor model of positive feelings and negative feelings (χ2/df = 1.494, GFI = 0.925, RMSEA = 0.071, SRMR = 0.008, CFI = 0.985, NFI = 0.958, NNFI = 0.980, TLI = 0.980, IFI = 0.986). Significant correlations with PHQ-9 with DDPRQ-10 and both subscales were revealed (r = 0.293 ~ 0.333, *p* < .001), while DDPRQ-10 score also significantly correlated with doctors’ MBI score (r = -0.467, *p* < .001). The MIRT model of full scale and IRT models of both subscales showed high discrimination of all items (a = 2.30 ~ 10.18), and the test information within the range of low-quality relationship was relatively high.

**Conclusion:**

The Chinese version of DDPRQ-10 displayed satisfactory reliability and validity and thus was appropriate for measuring the DPR in Chinese medical settings.

## Introduction

The doctor-patient relationship (DPR) is a crucial aspect of healthcare delivery in China. With the rapid development of the healthcare system, the DPR has become increasingly complex and challenging. It was reported that from 2009 to 2018, 295 severe medical violence events were reported on social media, in which 362 doctors were injured and 24 were killed [1]. However, the DPR seems to have faced a turning point during the outbreak of coronavirus disease 2019 (COVID-19) in China [2], Since the start of 2020, countless doctors have been on the frontlines of the pandemic, earning immense appreciation from the majority of the public. Some studies indicated that doctor-patient relationships in China saw improvement and an increase in trust during COVID-19 [3]. However, doubts regarding this notion have since emerged. So it is high time to address the importance of the DPR and to improve the medical environment. The DPR is a crucial factor in determining the quality of healthcare services, patient satisfaction, communication effectiveness, medical costs, and treatment outcomes. A positive DPR can significantly improve patient satisfaction and reduce medical costs [4, 5], and improving the DPR can lead to better health outcomes [5]. Therefore, research on DPR is not only helpful in improving medical quality and safeguarding patient rights, but also in optimizing the utilization of medical resources, reducing medical costs, improving doctor-patient communication, and promoting medical progress. To establish a healthy and stable DPR has become an urgent task facing the medical field, especially in China where there is an insufficiency of medical resources.

The commonly used scales to study DPR include the Patient-Doctor Relationship Questionnaire (PDRQ-9/PDRQ-18), the Consultation Satisfaction Questionnaire (CSQ), and the Doctor-Patient Relationship Questionnaire (DP − RQ). Most of the measurements of DPR were reported by patients to reveal their demands and comprehension. However, this simplistic view based on medical technology overlooks the fact that the interaction between doctors and patients is influenced by the attitudes of both parties. Various complex factors shape the behaviors of medical practitioners, who may not always adhere to clinical guidelines. Similarly, certain patient demands may be seen as irrational by doctors. Consequently, it is crucial to comprehend how medical doctors perceive and manage their relationship with patients [6]. Since the reliability and validity of the Chinese version of PDRQ-9 have been confirmed, which provided insights into patients, we further translated and examined the psychometric properties of the Chinese version of DDPRQ-10 [7]. The Difficult Doctor-Patient Relationship Questionnaire (DDPRQ-10), developed by Hahn et al., is widely used as a tool for assessing DPRs from the perspective of doctors in international medical surveys. The DDPRQ-10 is a simplified version of the 30-item DDPRQ, designed to evaluate DPRs from the doctors’ perspective [8]. The original full questionnaire was designed to identify patients with treatment difficulties, while the simplified version is widely used to assess the quality of DPRs perceived by primary care physicians [9]. Multiple studies have evaluated the reliability and validity of the questionnaire, and the results show that the DDPRQ-10 is a tool with good internal consistency and test–retest reliability [10, 11], which can effectively identify problems in DPRs, especially with regard to assessing patients’ emotional states and attitudes. Its reliability and validity have also been cross-culturally tested. In clinical applications, the DDPRQ-10 has been widely used in research on DPRs, doctor-patient communication training, and resolution of doctor-patient disputes, among other practical scenarios. It can help doctors better understand patients’ psychological states and improve doctor-patient communication and relationships [11–13]. This study aims to evaluate the reliability and validity of the Chinese version of the DDPRQ-10, providing an effective measurement tool for domestic DPR assessment from the perspective of doctors.

## Methods and materials

### Study design and participants

We conducted a descriptive, cross-sectional study to assess the psychometric properties and validation of the DDPRQ-10 scale. The sample size was calculated to fulfill the recommended 1:10 ratio of the number of items to the number of participants [14]. Between November 2022 and March 2023, a total of 38 responsible residents of 120 hospitalized patients were recruited from the neurology, gastroenterology, endocrinology, cardiology, obstetrics, and gynecology wards of Peking Union Medical College Hospital in China. The study evaluated inpatients aged 15 years or older who had been hospitalized for more than 24 hours and were able to read and sign the informed consent form. Patients with language barriers, limited writing skills, cognitive impairment/organic brain disorder/dementia, psychosis, and acute suicidal tendencies were excluded. All participants, including residents and patients, were informed of the study procedures, data collection, anonymization of personal data, and electronic informed consent with valid electronic signatures. For participants under 18 years old, additional informed consent from a parent was required.

Investigators, who were uniformly trained psychiatrists or graduate students of psychiatry, informed all participants about the investigation. After obtaining informed consent, participants received a QR code to scan and then filled in the questionnaires using their own mobile phones. Investigators were available to offer help if any incomprehension occurred. A total of 122 questionnaires were collected, and 2 invalid questionnaires were excluded due to unidentifiable information provided. The ethics committee of Peking Union Medical College Hospital approved the study, with the assurance that data would be reported anonymously in aggregate form.

### Measurements

#### Chinese version of the DDPRQ-10

The Chinese version of DDPRQ-10 consists of 10 items and is a medical evaluation scale used to assess the quality of DPRs from the perspective of doctors. It uses a 6-point scoring system ranging from 0 (not at all) to 5 (a great deal), with 7 items being reverse-scored. The total score ranges from 0 to 60, with higher scores indicating worse DPRs as perceived by the doctor. Previous studies have shown that the Cronbach's alpha coefficient for the English version of DDPRQ-10 is 0.779–0.88 [9, 15, 16]. The validity of the DDPRQ-10 has been supported by studies that have demonstrated patients who are perceived to have difficult doctor-patient relationships are often found to have psychiatric symptoms or disorders [8, 9, 17, 18], a history of childhood abuse [19], or an insecure attachment [20].

In this study, the Chinese version of the questionnaire was developed through a forward–backward translation process [21, 22] authorized by the scale's authors. The English version was translated into Chinese by 5 bilingual psychiatrists after discussion, and then independently back-translated into English by a senior psychiatrist with overseas experience. The back-translated version was compared with the original version by a native English speaker, who gave oral feedback on the alignment of the two versions. All authors then revised the translated version considering the feedback provided by the native English speaker. Ultimately, a consensus was reached among all authors, leading to the final version of the scale, with the items and scoring system remaining consistent with the original version.

#### Validation instruments

Referring to the original research by Hahn and his colleagues to assess the validity of the English version of DDPRQ-10 and the conclusion that the difficult DPR was partly due to physical symptoms and mental disorders of patients [9], our study introduced measurements of depressive symptoms to evaluate the convergent validity of the Chinese version scale, by looking for a significant correlation between DDPRQ-10 ratings and depressive symptoms. To assess divergent validity, we examined correlations with theoretically unrelated constructs such as patients' age.

We used the Patient Health Questionnaire Depression Scale-9 item (PHQ-9) to evaluate patients' depressive symptoms [23]. The PHQ-9 was developed according to the diagnostic criteria of major depressive disorders (MDD) following the Diagnostic and Statistical Manual of Mental Disorders, Fourth Edition (DSM-IV) [24]. Participants rated perceived symptom burden during the past two weeks between 0 (not at all) and 3 (nearly every day), resulting in a total score ranging from 0 to 27. The Chinese version of PHQ-9 has been validated in numerous studies, with a generally accepted cut-off score of 10 [25–27], and the internal consistency of the PHQ-9 for this study was high (Cronbach's α = 0.88) [27].

Previous studies have also suggested that there was a significant correlation between physician burnout, self-efficacy, and the DPR. Higher levels of burnout indicated that doctors needed to extend their working hours to maintain the time and energy allocated to each patient, often indicating lower-quality DPRs [28]. On the other hand, higher levels of self-efficacy meant that doctors could make clinical decisions more independently and confidently, which helped to improve the quality of DPRs [29, 30]. Therefore, we also included the Maslach Burnout Inventory [31, 32] to evaluate the validity of the DDPRQ-10 scale.

The Maslach Burnout Inventory (MBI) [31] was a medical evaluation scale that assessed the level of burnout among doctors. It included three subscales: emotional exhaustion, depersonalization, and personal accomplishment, with 9, 5, and 8 items respectively. The personal accomplishment subscale was reverse scored, while the others were positively scored. Each item was scored on a 5-point scale ranging from 0 (never) to 4 (always), with higher total scores indicating higher levels of burnout.

#### Sociodemographic questionnaire

Each patient’s information regarding age, gender, residence, family status, family income, level of education, and essential worker status was gathered through a demographic questionnaire.

### Statistical analysis

To validate the Chinese version of DDPRQ-10, the following methods were used with a statistical significance criterion of *P* < 0.05:

- a) Descriptive statistics: Continuous variables and categorical variables were described using mean ± standard deviation (mean ± SD) and numbers with percentages [n (%)] respectively. Student's t-tests and one-way ANOVA tests were used to compare the differences in DDPRQ-10 scores among different groups.
- b) Item analysis: Corrected item-total correlations were calculated to measure the strength of the relationship between each item and the total score of the scale. A significant correlation coefficient larger than 0.4 was suggested as satisfactory [33].
- c) Structural validity: The sample was randomly split in half to perform exploratory factor analysis (EFA) and confirmatory factor analysis (CFA) using IBM SPSS 20.0 and AMOS 27 respectively. Before conducting the EFA, data suitability and sampling adequacy were checked using the Kaiser–Meyer–Olkin (KMO) value and Bartlett's test of sphericity. During the principal components analysis, factors with an eigenvalue larger than 1 were extracted. A total factor loading of more than 60% was considered acceptable [34]. Secondly, a confirmatory factor analysis (CFA) [estimation method = diagonal weighted least square] was carried out. Acceptable model fit was defined by a standardized root mean square residual (SRMR) [35] value ≤ 0.08, a root-mean-square-error of approximation (RMSEA) [36] value ≤ 0.10, with comparative fit index (CFI) [37] and Tucker-Lewis index (TLI) [38] values ≥ 0.90.
- d) The study calculated Pearson correlation coefficients between patients' age, PHQ-9 rating, and total scores of MBI, and DDPRQ-10, as well as their subscales, to assess convergent and divergent validity. We hypothesized that DDPRQ-10 scores would significantly correlate with PHQ-9 ratings and MBI scores, thus supporting the scale's convergent validity.
- e) To evaluate the internal consistency of the Chinese version of DDPRQ-10 and its subscales, the study used Cronbach's α. A Cronbach's α coefficient greater than 0.70 was considered sufficient [39].
- f) In order to conduct classical item response theory(IRT) analysis, the scale's construct must meet the unidimensional criterion [40]. If the factor analysis in step c) reveals that the Chinese version of DDPRQ-10 contains more than one dimension, multi-dimension item response theory (MIRT) analysis [41] would be conducted using IRTPRO 6.0 software following the Samejima graded response model [42]. The MIRT discrimination and intercept parameters of each item would be computed based on the multidimensional model constructed in step c), and the correlation θ between each potential dimension would be calculated along with its 95% confidence interval. If the upper limits of all confidence intervals were less than 1, it would indicate that the potential dimensions do not completely overlap, and the data is consistent with a multidimensional model rather than a unidimensional model. DDPRQ-10 would then be divided into subscales based on the multidimensional model, and the unidimensionality assumption would be tested for each subscale using factor analyses. IRT analysis with a fitted Samejima graded response model would be conducted for each subscale to estimate the discrimination and intercept parameters of every item. Next, plots of item infit and outfit statistics and person-item maps would be drawn to evaluate item fit as well as person fit. Information curves would be drawn for each item and subscale. Lastly, with regard to influences of gender, measurement invariance was represented by differential item functioning (DIF) based on the Mantel DIF contrast test with the Bonferroni significant level correction. We checked each item to ascertain whether they performed differently in subgroups (i.e., females vs. males).

## Results

### Descriptive statistics

We recruited 120 patients with an average age of 52.55 ± 16.83 years who completed the PHQ-9 questionnaires. Additionally, 38 responsible residents also completed the DDPRQ-10 rating for these patients. 49 (39.2%) of all these patients were female, with the average DDPRQ-10 score 33.45 ± 4.44. The sociodemographic characteristics were presented in Table 1. There was no significant difference in DDPRQ-10 ratings among patients based on age, place of residence, educational level, family status, family income, or essential worker status. However, the DDPRQ-10 scores of female patients were significantly higher than those of male participants.

**Table 1:** Sociodemographic characteristics of total sample

Characteristics	N	Proportion (%)	Mean	SD	*P*-value
Age					0.163^§^
≤ 20	6	5.0%	3.17	2.32	
21–40	24	20.0%	10.79	11.95	
41–60	57	47.5%	9.61	10.08	
> 60	33	27.5%	6.39	8.62	
Gender					0.095^†^
Male	71	60.8%	9.68	10.64	
Female	49	39.2%	6.74	8.67	
Residence					0.226^†^
City	95	79.2%	9.21	10.40	
Rural	25	20.8%	6.48	8.11	
Family status					0.982^§^
Single	16	13.3%	8.25	9.97	
Married	97	80.8%	8.76	9.93	
Divorced/Widowed	6	5.0%	8.33	13.53	
Other	1	0.8%	5.00	/	
Monthly family income					0.920^§^
4000–8000 RMB	15	12.5%	9.11	10.37	
More than 8000 RMB	45	37.5%	8.42	10.43	
Less than 4000 RMB	60	50%	8.13	7.23	
Essential Worker Status					0.807^§^
Employed/Student	62	51.7%	8.47	10.56	
Unemployed	25	20.8%	8.12	9.62	
Retired	25	20.8%	8.52	8.96	
Other	8	6.7%	12.00	10.94	
Education					0.197^§^
College preparatory	36	30%	9.26	8.86	
Elementary	34	28.3%	6.17	8.05	
University or higher	50	41.7%	10.00	11.70	

^†^ Student’s t-test

^§^ One-way ANOVA test

^**^ P < .01

### Item analysis

To assess the corrected item-total correlation coefficients, we performed a Pearson correlation analysis by comparing the score of each individual item in the DDPRQ-10 with the total score obtained by subtracting that item’s score. The results showed that all correlation coefficients ranged from 0.546 to 0.874, satisfying the requirement of being greater than 0.40 and revealing statistical significance. This suggests that all items in the scale demonstrate satisfactory consistency with the construct being measured.

### Structural validity analysis

We randomly divided the total sample into two halves, each of which contained 60 participants and a factor analysis was performed on the first half of samples to determine the number of factors. The KMO statistic was 0.784, indicating that factor extraction was appropriate based on the data. Bartlett's test of sphericity [χ^2^ [34] = 521.08, *P* < 0.001] also supported the suitability of the data for factor extraction. By principal component analysis with the varimax rotation method, the analysis yielded two common factors with eigenvalues above 1, explaining 76.00% of the variation. The factor loading of each item, presented in Table 2, indicated that items 2, 3, 4, 5, 6, 8, and 10 were classified as negative feelings, while items 1, 7, and 9 were classified as positive feelings. Most items had a loading above 0.7, except for item 7, which had a loading of 0.580.

**Table 2:** Results of item analysis and factor loadings

Item	Corrected Item-total Correlation	Loadings on Factor 1	Loadings on Factor 2
How much are you looking forward to this patient's next visit after seeing this patient today?	0.724^***^	0.251	0.841
How "frustrating" do you find this patient?	0.874^***^	0.883	0.307
How manipulative is this patient?	0.728^***^	0.849	0.201
To what extent are you frustrated by this patient's vague complaints?	0.824^***^	0.732	0.481
How self-destructive is this patient?	0.546^***^	0.883	0.083
Do you find yourself secretly hoping that this patient will not return?	0.572^***^	0.716	0.114
How at ease did you feel when you were with this patient today?	0.792^***^	0.241	0.906
How time consuming is caring for this patient?	0.751^***^	0.580	0.539
How enthusiastic do you feel about caring for this patient?	0.724^***^	0.124	0.932
How difficult is it to communicate with this patient?	0.807^***^	0.722	0.451

^*^P < 0.05, ^**^P < 0.01, ^***^P < 0.001

**Fig. 1:**
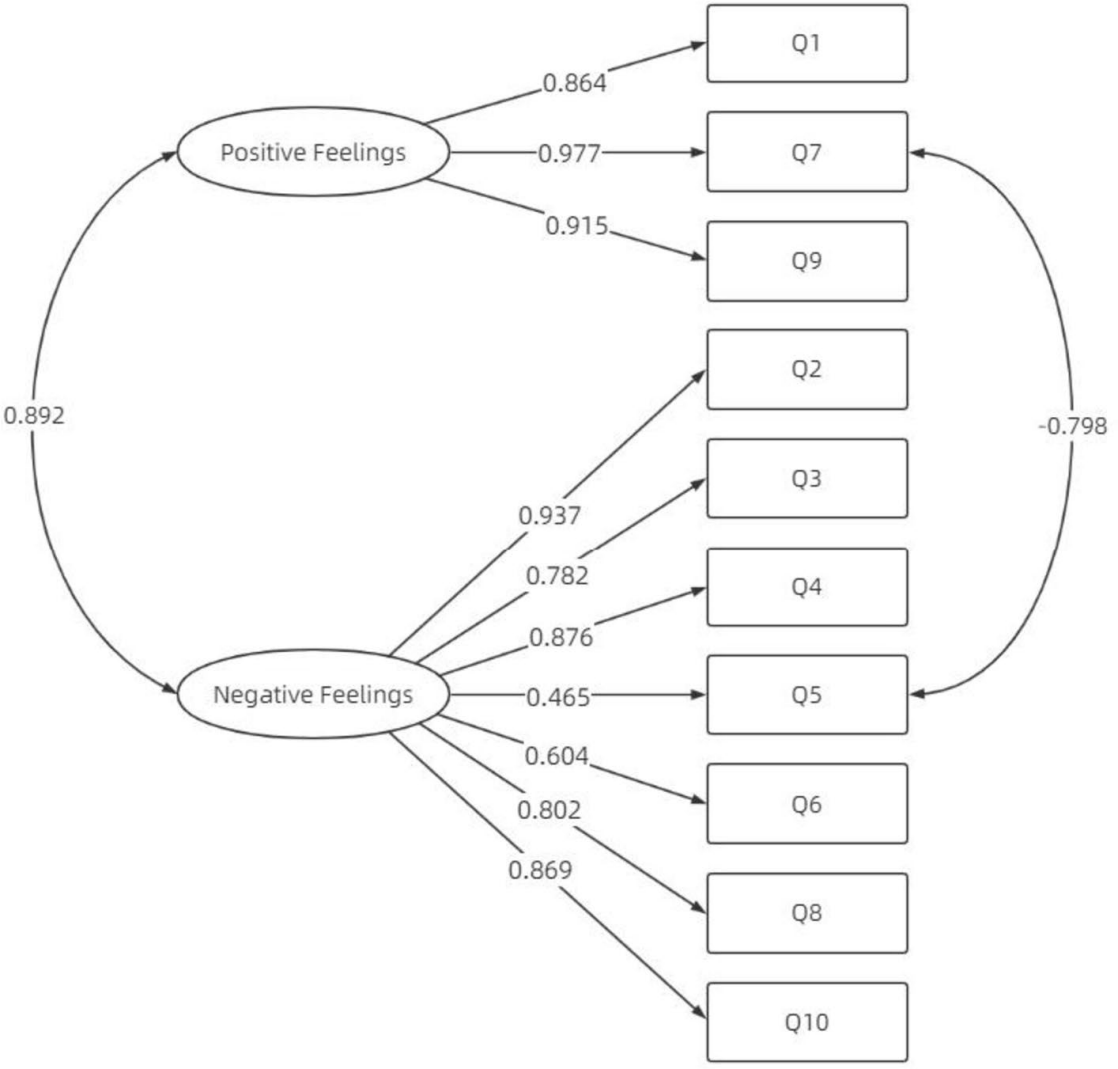
Factor structure of the difficult doctor-patient relationship questionnaire Chinese version

In the other random sample (*n* = 60), a confirmatory factor analysis with weighted least square estimation was conducted to test the modified two-factor model of negative and positive feelings. The analysis showed that the factor loading of each item in the model was above 0.4, as depicted in Fig. 1. Additionally, the model fit indexes indicated an excellent fit for the two-factor model (χ2/df = 1.340, RMSEA = 0.075, SRMR = 0.042, CFI = 0.979, TLI = 0.972), as shown in Table 3. For the finally established model, the Composite Reliability (CR) value and the Average Variance Extracted (AVE) value for each dimension were calculated. It was found that the CR value for the positive feelings dimension was 0.938, and the AVE value was 0.843. The CR value for the negative feelings dimension was 0.900, with an AVE value of 0.625. Both dimensions showed CR values greater than 0.8 and AVE values exceeding 0.5, indicating that the scale possesses good construct validity. In conclusion, the results confirmed the suitability of the modified two-factor model to the data.

**Table 3:** Model fit indices of different models

Items on the DDPRQ-10	*χ*^2^	*df*	*P*	RMSEA	CFI	TLI	SRMR
One-factor model	92.451	35	< .0001	0.165	0.895	0.865	0.051
Two-factor model	59.275	34	.0046	0.111	0.954	0.939	0.044
**Two-factor model (modified)**	**44.214**	**33**	**.0919**	**0.075**	**0.979**	**0.972**	**0.042**

Notes: The chosen model is shown in bold

*Abbreviations*: *CFI* Comparative fit index, *DDPRQ* Difficult Doctor-Patient Relationship Questionnaire, *RMSEA *Root-mean-square-error of approximation, *SRMR* Standardized root mean square residual, *TLI* Tucker-Lewis index

### Correlational analysis

The total score of DDPRQ-10, as well as the scores of positive and negative feelings, displayed positive correlations with the score of PHQ-9 (r = 0.329, 0.333, and 0.293, respectively, *p* < 0.001) as presented in Table 4. This suggests that higher scores on the DDPRQ-10 and its subscales are associated with higher scores on the PHQ-9. Furthermore, the total score of DDPRQ showed a significant negative correlation with the corresponding resident doctors' MBI score (r = -0.467, *p* < 0.001). This indicates that resident doctors experiencing greater career burnout may have a worse doctor-patient relationship. These findings support the correlations of the DDPRQ-10 questionnaire and factors related to doctor-patient relationship from both patients and doctors’ perspective.

**Table 4:** Descriptive statistics and correlation coefficients between variables

**Item**	$\overline{\mathcal{X} }$** ± s**	**DDPRQ-10**	**DDPRQ-PF**	**DDPRQ-NF**	**PHQ-9**
DDPRQ-10	8.64 ± 10.0	-			
DDPRQ-PF	2.81 ± 3.53	0.856***	-		
DDPRQ-NF	5.83 ± 7.21	0.967***	0.698***	-	
PHQ-9	5.06 ± 0.52	0.329**	0.333**	-0.293**	-

*DDPRQ-10* Difficult Doctor-Patient Relationship Questionnaire Chinese Version, *DDPRQ-NF* Negative feelings subscale of DDPRQ-10 Chinese version, *DDPRQ-PF* Positive feelings subscale of DDPRQ-10 Chinese version, *PHQ-9* Patient Health Questionnaire Depression Scale-9 item, PHQ-9; ^**^P < 0.01; ^**^P < 0.001

### Reliability analysis

The DDPRQ-10 questionnaire had a Cronbach's α coefficient of 0.931 for the full scale, 0.926 for the positive feelings’ subscale and 0.909 for the negative feelings’ subscale. The unequal-length Spearman-Brown split-half reliability for the full scale was 0.917, indicating that the scale is reliable.

### Analysis based on item response theory

We performed MIRT (Multidimensional Item Response Theory) analysis on the Chinese version of the DDPRQ-10 questionnaire due to the identification of two underlying dimensions in the factor analyses. We used the Samejima graded response model to estimate discrimination parameters (a) and difficulty parameters (b) for each item in the full scale (refer to Table 5). The discrimination parameters ranged from 2.30 to 10.18, all of which were considered to be very high, indicating that the items were effective in discriminating between different levels of the construct. The MIRT model revealed a correlation (θ) of 0.85 between the two dimensions, with a 95% confidence interval of [0.77, 0.93]. The fact that the upper limit was less than 1 suggests a high correlation but not complete overlap between the two dimensions. These findings supported a two-factor model rather than a unidimensional model for the Chinese version of the DDPRQ-10 questionnaire.

**Table 5:** Item content of DDPRQ-10 full scale and MIRT item parameter estimates

Items on the DDPRQ-10	a1	a2	b1	b2	b3	b4	b5
How much are you looking forward to this patient's next visit after seeing this patient today?	4.86 ± 0.95	-	8.73 ± 1.26	8.3 ± 1.20	6.04 ± 0.96	4.39 ± 0.82	0.6 ± 0.56
How "frustrating" do you find this patient?	-	8.24 ± 1.59	15.82 ± 2.59	13.94 ± 2.20	8.55 ± 1.35	6.9 ± 1.16	4.27 ± 0.96
How manipulative is this patient?	-	3.41 ± 0.73	7.69 ± 1.24	4.06 ± 0.69	2.72 ± 0.57	1.66 ± 0.5	-
To what extent are you frustrated by this patient's vague complaints?	-	5.01 ± 1.01	11.21 ± 1.9	8.44 ± 1.28	5.72 ± 0.96	3.89 ± 0.78	1.97 ± 0.65
How self-destructive is this patient?	-	2.84 ± 0.68	7.55 ± 1.39	4.36 ± 0.76	4.05 ± 0.72	2.8 ± 0.61	-
Do you find yourself secretly hoping that this patient will not return?	-	2.3 ± 0.5	4.33 ± 0.60	4.08 ± 0.57	2.77 ± 0.45	2.34 ± 0.42	0.97 ± 0.34
How at ease did you feel when you were with this patient today?	10.18 ± 1.47	-	18.05 ± 2.19	15.65 ± 1.62	11.44 ± 1.30	8.09 ± 1.02	2.43 ± 0.96
How time consuming is caring for this patient?	-	2.74 ± 0.52	5.46 ± 0.73	4.8 ± 0.64	2.58 ± 0.44	1.17 ± 0.37	-0.25 ± 0.34
How enthusiastic do you feel about caring for this patient?	5.60 ± 1.11	-	10.68 ± 1.64	9.45 ± 1.42	5.54 ± 0.97	3.89 ± 0.83	0.73 ± 0.62
How difficult is it to communicate with this patient?	-	4.19 ± 0.88	9.88 ± 1.69	8.19 ± 1.27	4.86 ± 0.84	3.97 ± 0.76	1.72 ± 0.58

Principal component analysis was used to assess the assumption of unidimensionality for each subscale. For the positive feeling subscale, the first factor eigenvalue was 2.615, accounting for 87.17% of the variation, whereas the second eigenvalue was 0.243, less than one-third of the first eigenvalue. Similarly, for the negative feeling subscale, the first factor eigenvalue was 4.678, accounting for 66.82% of the variation, and the second eigenvalue was 0.697, also less than one-third of the first eigenvalue. These results indicated that both subscales met the assumption of unidimensionality and were suitable for IRT analysis. The discrimination parameters for the items in both subscales ranged from 2.36 to 10.23 (refer to Table 6), indicating their effectiveness in differentiating responses.

**Table 6:** Item content of DDPRQ-10 subscales and IRT item parameter estimates

Subscale	Items on the DDPRQ-10	a1	c1	c2	c3	c4	c5
Positive Feelings	How much are you looking forward to this patient's next visit after seeing this patient today?	4.88 ± 0.95	0.1 ± 0.14	0.89 ± 0.16	1.24 ± 0.18	1.72 ± 0.24	1.81 ± 0.26
	How at ease did you feel when you were with this patient today?	7.85 ± 3	0.22 ± 0.14	0.78 ± 0.15	1.12 ± 0.17	1.58 ± 0.21	1.87 ± 0.27
	How enthusiastic do you feel about caring for this patient?	6.13 ± 1.46	0.11 ± 0.14	0.67 ± 0.15	0.97 ± 0.17	1.69 ± 0.24	1.93 ± 0.29
Negative Feelings	How "frustrating" do you find this patient?	10.23 ± 10.86	0.48 ± 0.23	0.8 ± 0.25	1 ± 0.27	1.69 ± 0.5	1.91 ± 0.5
	How manipulative is this patient?	3.41 ± 1.08	0.45 ± 0.25	0.77 ± 0.3	1.18 ± 0.38	2.33 ± 0.71	-
	To what extent are you frustrated by this patient's vague complaints?	4.51 ± 1.52	0.36 ± 0.22	0.75 ± 0.28	1.14 ± 0.36	1.74 ± 0.54	2.55 ± 0.68
	How self-destructive is this patient?	2.83 ± 0.8	0.96 ± 0.33	1.42 ± 0.41	1.53 ± 0.44	2.88 ± 0.77	-
	Do you find yourself secretly hoping that this patient will not return?	2.36 ± 0.70	0.4 ± 0.25	0.99 ± 0.37	1.18 ± 0.43	1.76 ± 0.60	1.87 ± 0.62
	How time consuming is caring for this patient?	2.55 ± 0.67	-0.11 ± 0.18	0.41 ± 0.25	0.95 ± 0.34	1.8 ± 0.54	2.07 ± 0.6
	How difficult is it to communicate with this patient?	3.63 ± 1.22	0.39 ± 0.23	0.94 ± 0.33	1.17 ± 0.37	2.08 ± 0.67	2.7 ± 0.77

**Fig. 2:**
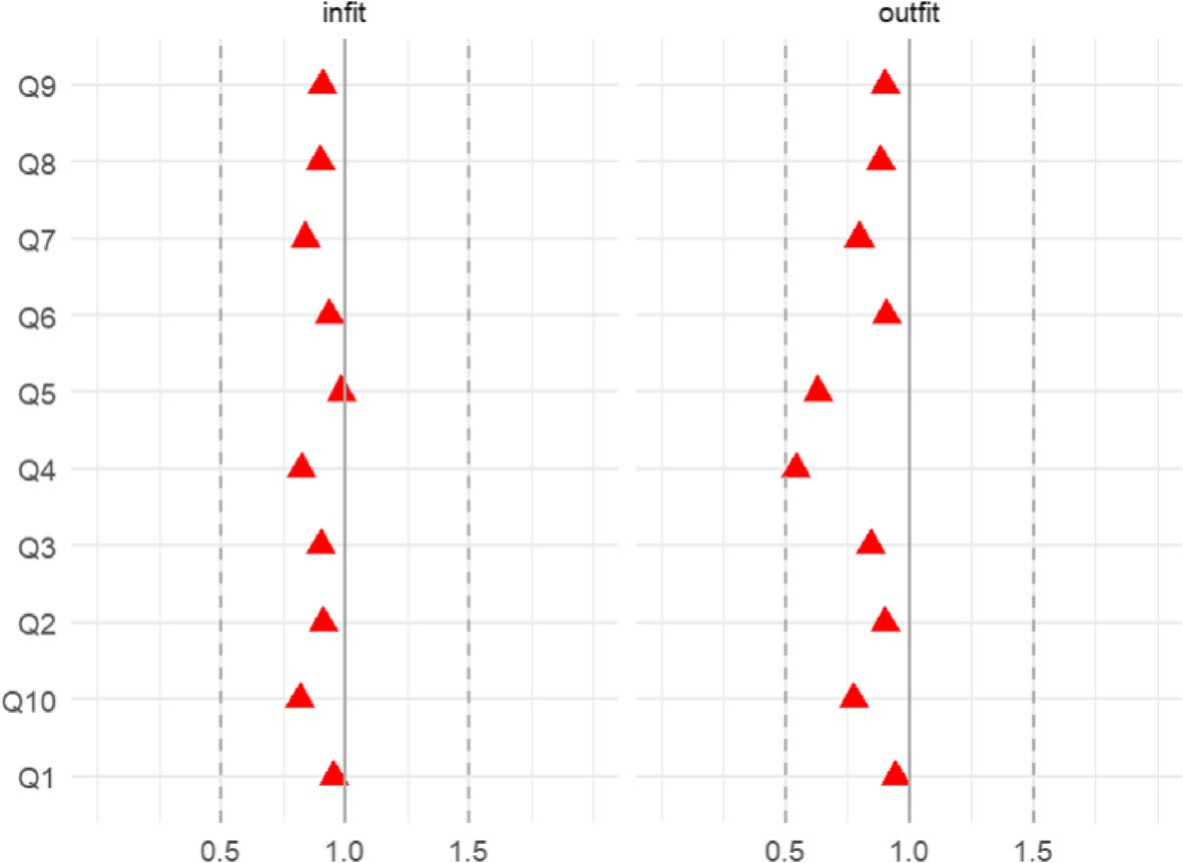
Item infit and outfit statistics for the DDPRQ-10

**Fig. 3:**
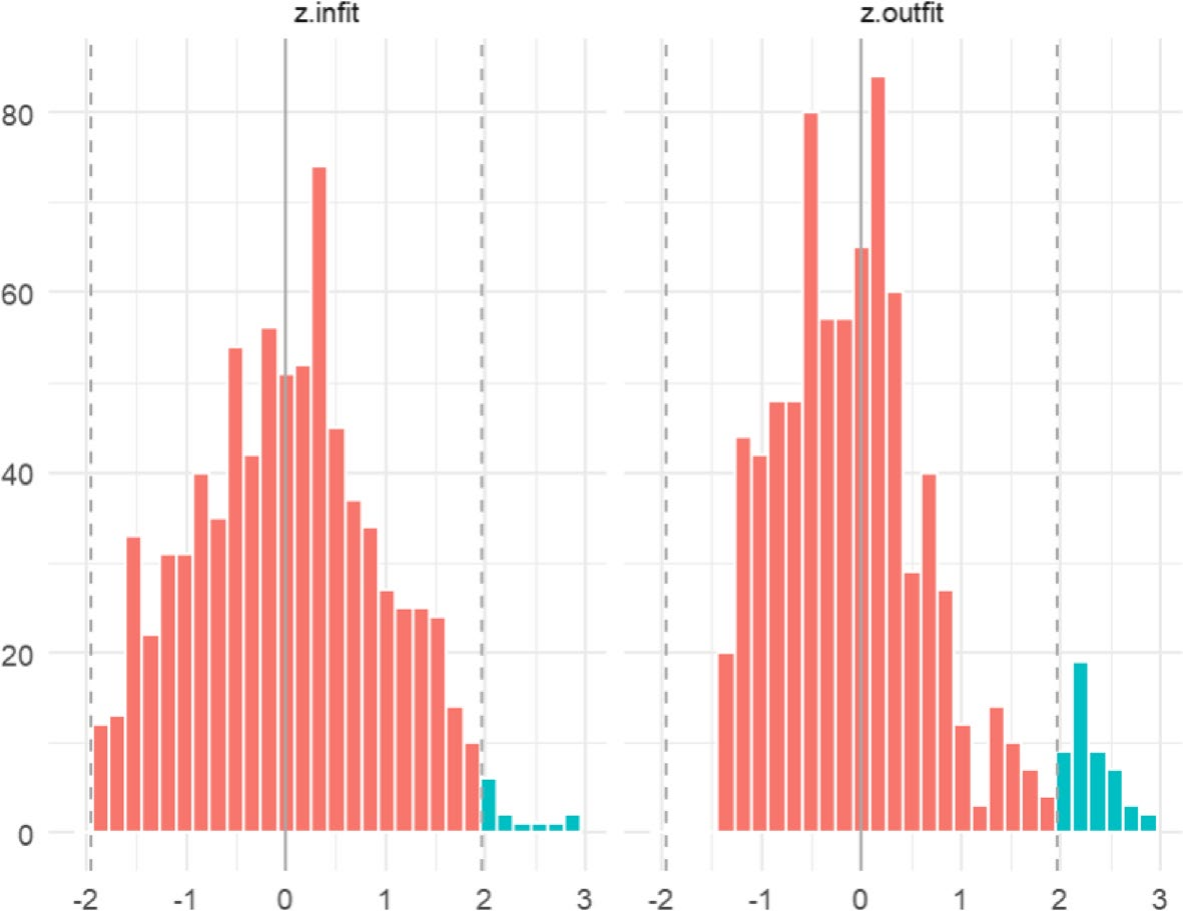
Person infit and outfit statistics for the sample on DDPRQ-10

The item infit and outfit statistics were shown in Fig. 2. The non-standardized values should be between 0.5 and 1.5 to not be degrading. Although Q4 has a relatively lower fit (outfit value close to 0.5), all items met the standard. The person infit and outfit statistics were shown in Fig. 3. If less than 5% of the respondents have higher or lower infit and outfit values than 1.96 and -1.96, the person fit would be considered to be good. In the current sample, only 1.63% of the patients ranged outside on the infit index, 6.13% of the patients ranged outside on the outfit index. Despite a small number of patients exceeding the given standard range in outfit statistics, the overall infit and outfit statistics of the scale are considered acceptable.

**Fig. 4:**
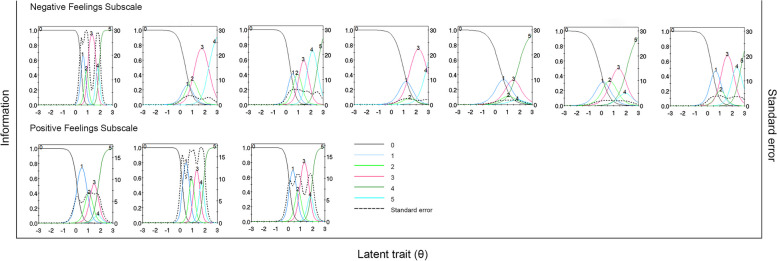
Item characteristic curves and item information curves of items in the DDPRQ-10

**Fig. 5:**
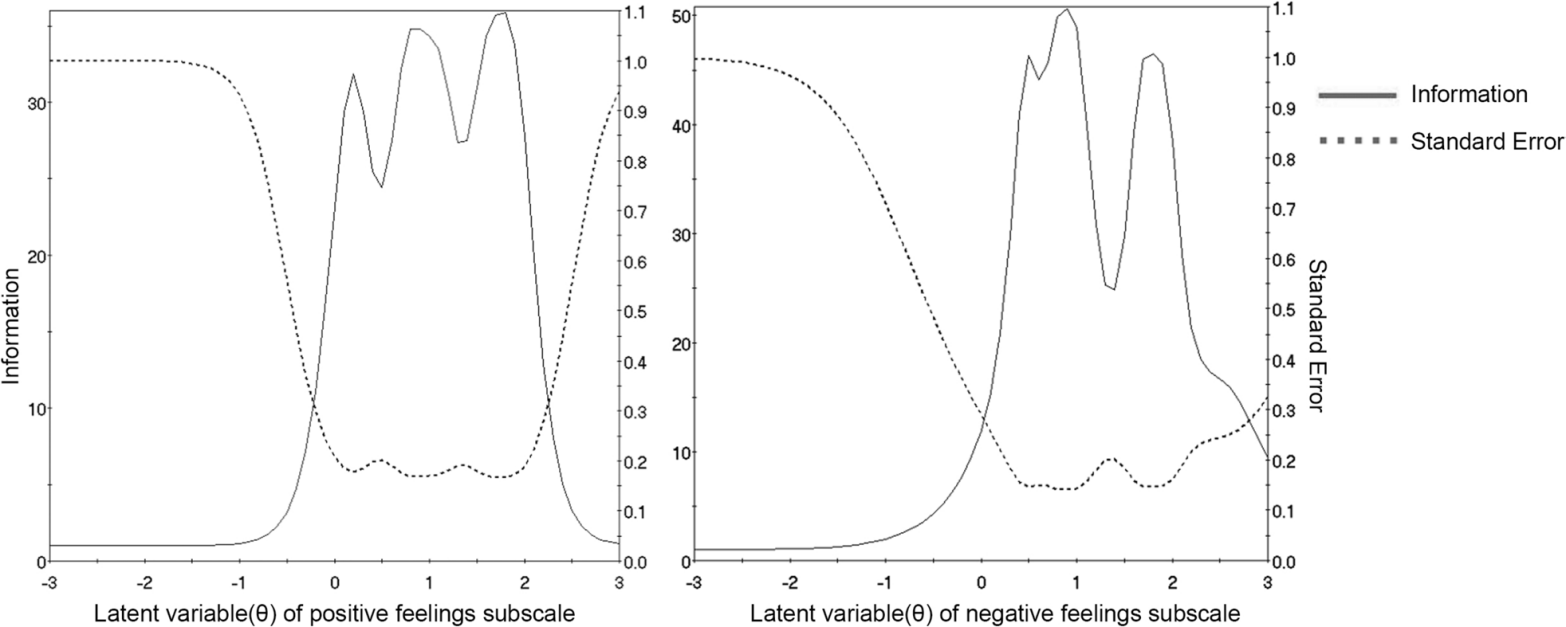
Item information curves of DDPRQ-10 subscales

Item level information clarifies how well each item contributes to score estimation precision with higher levels of information leading to more accurate score estimates. In Fig. 4, the category characteristics curves and item information curves for all items were presented. It can be clearly seen that items offer most information on higher theta levels, which means more information were offered for patients with more difficulties in doctor-patient relationship. Moreover, the concept of “information” can also be applied to the entire scale. Figure 5 showed the item information curves for the two subscales. We see that the scale is very good at estimating theta scores between 0 and 2.5, but has less precision at estimate theta scores of other ranges. The analysis of the information curves indicated that the scale demonstrated robust performance in identifying and screening patients who are encountering difficulties or conflicts within the doctor-patient relationship. Conversely, the scale's efficacy appeared to be diminished when applied to patients who are experiencing a harmonious doctor-patient relationship. This indicates that the scale is particularly useful for capturing and assessing situations involving more challenging or conflicted relationships.

Finally, to assess the validity of the DDPRQ scores with respect to gender, DIF analysis was performed using the Mantel DIF contrast test with the Bonferroni significant level correction according to the number of comparisons (0.05/9) as recommended by Linacre [43]. The DIF analysis revealed that all IPS items functioned similarly for both gender groups in the current sample (DIF contrast was less than the cut-off point of 0.64 and Mantel–Haenszel probabilities for all items were above 0.05). All items on the DDPRQ were thus concluded to be equitable to both male and female individuals.

## Discussion

In this study, doctors were recruited to evaluate inpatients from a general hospital in China to explore the reliability, validity and psychometric characteristics of DDPRQ-10 Chinese as an instrument measuring DPR in Chinese hospitals. The findings indicated that the Chinese version of the DDPRQ-10 was consistent with a modified two-factor model of positive and negative feelings, demonstrating satisfactory internal consistency, discrimination, reliability, and validity. The questionnaire was found to be useful in identifying and screening DPRs that were more challenging or conflicted, making it a viable option for evaluating DPRs in Chinese culture. Besides its psychometric properties, the research also revealed some findings that have not been reported previously and require further investigation in cross-culture circumstances.

Firstly, this study confirmed that the Chinese version of the DDPRQ-10 questionnaire conformed to the adjusted two-factor structure model, which included two factors of negative feelings and positive feelings. These factors respectively represented the negative emotions that doctors felt in the DPR, such as difficulties, frustration, and avoidance, as well as positive emotions such as relaxation, enthusiasm, and anticipation. In Hahn's original study of the DDPRQ-10 [8, 9, 17], the questionnaire could be divided into subscales including subjective experiences of doctors, objective evaluations of patient behavior, and symptoms. Another study on the DPR divided the DPPRQ-10 questionnaire into three dimensions: negative personality traits of patients, communication difficulties, and negative emotional responses of doctors [10]. These studies had similar dimensions, all of which reflected the importance of patient traits, doctors' subjective experiences, and effective communication in the DPR. However, the two dimensions reflected in this study mainly revolved around the subjective experiences of doctors in the diagnosis and treatment process. This difference may be related to different cultures and medical backgrounds. Due to the insufficient medical resources in China, the DPR is mostly dominated by doctors to increase efficiency, so doctors' experiences and coping strategies play a major role in the DPR.

Secondly, the study used statistical methods based on item response theory to assess the psychometric properties of DDPRQ-10 for the first time. The results indicated that each item had satisfactory discrimination in both the multidimensional IRT model of the full scale and unidimensional IRT models of the two subscales. The item information curves showed that DDPRQ-10 was more effective in providing information for difficult DPRs, but not for patients with good DPRs. Therefore, DDPRQ-10 is more suitable for screening and risk assessment of difficult DPRs, and can provide sensitive indicators for intervention studies on such relationships. Future research should investigate whether this information function model is consistent in other cultural environments and explore ways to improve the measurement performance of DDPRQ-10 in measuring good DPRs.

Thirdly, the significant correlation of DDPRQ-10 Chinese scale with PHQ-9, and MBI scale was reported. This study proved a correlation between the difficulty of DPRs and the level of depression in patients through correlation analysis with the PHQ-9 scale, which was consistent with previous study. Hahn et al. conducted the original study on the DDRPQ-10 [9], showing that patients with physical symptoms and psychological disorders were more likely to be identified as having difficult DPRs. In addition, the study by Jackson et al. [44] also confirmed that mental illnesses such as depression, anxiety, or personality disorders usually indicate poor DPRs, especially for patients diagnosed with more than four mental illnesses, with a 100% identification rate for difficult DPRs. Furthermore, the main predictive factors for poor DPRs include the presence of five or more physical symptoms, poor functional status, threatening and aggressive personality, failure to meet expectations, and high frequency of medical visits [44]. This result once again demonstrated the importance of identifying patients with serious mental and psychological comorbid such as depression, and incorporating this factor into the DPR for establishing a good DPR among non-psychiatric physicians.

In addition, this study also found a significant correlation between difficult DPRs and physician burnout. A large body of researches have similarly shown a bidirectional relationship between burnout and difficult DPRs, with physician burnout being one of the contributing factors to poor DPRs [45], while difficult DPRs can also exacerbate physician burnout [46, 47]. Therefore, in clinical practice, taking measures to alleviate physician burnout is crucial for shaping good DPRs.

There are also some limitations in this study. Firstly, due to the limited number of validated and widely used tools for evaluating DPRs and patient treatment satisfaction in Chinese, this study used the validated Chinese version of the PHQ-9 and MBI to explore possible correlations. In the future, more validated scales can be included in the patient’s evaluation to comprehensively assess the mental and personality traits of patients from the perspective of patient factors in DPRs. Secondly, the doctors included in this study were non-psychiatric specialists in general hospitals, and the sample size was relatively small, which may lead to selection bias. In the future, a larger sample of doctors from different departments, including surgical and non-surgical departments, can be included for scale measurement and analysis to further validate the robustness of its psychological measurement properties. Thirdly, the examination of the test–retest reliability was not yet conducted in this research, and the stability of the DDPRQ-10 across time needs to be tested in future studies.

## Conclusion

The reliability and validity of the Chinese version of DDPRQ-10 were found to be satisfactory, indicating that it can effectively measure DPR in Chinese medical settings. This tool could be utilized for assessing DPR in medical settings in China, allowing for effective measurement and monitoring of the doctor-patient relationship, leading to potential improvements in healthcare quality and patient satisfaction.

## Data Availability

The datasets analyzed in this article are not publicly available. Requests to access the datasets should be directed to LS, shill@pumch.cn.

## Abbreviations

CFA: Confirmatory factor analysis
CFI: Comparative fit index
COVID-19: Coronavirus disease 2019
DDPRQ-10: Difficult Doctor-Patient Relationship Questionnaire
DPR: Doctor-patient relationship
DSM-IV: Diagnostic and Statistical Manual of Mental Disorders, Fourth Edition
EFA: Exploratory factor analysis
IRT: Item response theory
KMO value: Kaiser–Meyer–Olkin value
MBI: Maslach Burnout Inventory
MDD: Major depressive disorders
MIRT: Multi-dimension item response theory
PHQ-9: Patient Health Questionnaire Depression Scale-9
QR Code: Quick response code
RMSEA: Root-mean-square-error of approximation
SRMR: Standardized root mean square residual
TLI: Tucker-Lewis index
